# Etiology of Ulcerative Dermal Necrosis (UDN) in Brown Trout (*Salmo trutta* Morpha *trutta*)—Preliminary Results

**DOI:** 10.3390/pathogens13030251

**Published:** 2024-03-15

**Authors:** Marek Matras, Magdalena Stachnik, Anna Kycko, Magdalena Wasiak, Ewa Paździor, Joanna Maj-Paluch, Ewa Borzym, Michał Reichert

**Affiliations:** 1 Department of Fish Diseases, National Veterinary Research Institute, 57 Partyzantów Avenue, 24-100 Pulawy, Poland; marek.matras@piwet.pulawy.pl (M.M.); magdalena.stachnik@piwet.pulawy.pl (M.S.); ewa.pazdzior@piwet.pulawy.pl (E.P.); joanna.maj@piwet.pulawy.pl (J.M.-P.); ewa.borzym@piwet.pulawy.pl (E.B.); 2 Department of Pathology, National Veterinary Research Institute, 57 Partyzantów Avenue, 24-100 Pulawy, Poland; anna.kycko@piwet.pulawy.pl (A.K.); magdalena.wasiak@piwet.pulawy.pl (M.W.)

**Keywords:** pathogenesis, spawning migration, salmonids, pathology, skin lesions

## Abstract

Every year, ulcerative dermal necrosis (UDN) affects salmonids that spend most of their lives in the sea during their migration to the rivers of northern Poland to spawn. The clinical form of the disease manifests itself in ulcerative skin lesions, which lead to significant weakening of the fish and, in most cases, result in their death. This study was carried out on samples taken from sea trout in the Słupia River in northern Poland. In order to identify the pathogen, experiments on the transmission of the disease were carried out, and additional histopathological, microbiological and electron microscopic examinations were performed. As a result of these studies, it was possible to experimentally transfer the disease from sick to healthy fish. The results indicate a complex etiology of the disease (lack of a clearly defined pathogen), in which the change in the environment from salty to freshwater triggers the related changes in skin physiology, which are the main causes of increased susceptibility to the development of the disease.

## 1. Introduction

The first registered epizootic episode of this disease was defined in 1877 and then referred to as salmonid disease [1]. Both sea trout (*Salmo trutta* morpha *trutta*) and salmon (Salmo salar) are anadromous fish belonging to the same genus and are considered to be two of the most economically, biologically and socially important fish species, ensuring the existence of commercial and recreational fishing along the Atlantic and Baltic coasts. Sea trout, like other anadromous fish, also play an important role in maintaining the overall balance of ecosystems. In the Baltic region, sea trout breed naturally in 25 rivers, and some of these populations are supported by restocking [2]. In Poland, sea trout are found in many Pomeranian rivers and in the Odra and Vistula basins. The total catches of sea trout (coastal, river and sea) in the years 1972–1994 fluctuated to a small extent and ranged from 50 to 200 tons, with one exception in 1990, when it was about 500 tons [3]. From 1995, the catches increased steadily, reaching as many as 863 tons in 2002. After 2002, catches decreased systematically. Among the reasons for the steady decline in the catches of sea trout observed since 2002, UDN can be seriously considered. In particular, in 2007, a significant outbreak of UDN in the Pomeranian rivers was recorded [4]. The serious losses of sea trout caused by UDN threaten the preservation of the genetic resources of this valuable species. The most severe outbreak of the disease in Poland was observed in fish from the Słupia River, where 74.7% of the individuals caught in 2007 showed clinical symptoms of UDN. Clinical signs suggested an infectious (fungal/bacterial and/or viral) etiology of the disease [4]. However, what has been usually seen, i.e., dramatically infested fish covered with ulcers and mold, is only the last stage of the disease. We do not know what initiates the pathological process. Solving this puzzle is the key to understanding the pathogenesis of the disease. So far, no virus has been detected as a UDN-related pathogen. However, more than 40 years ago, it was shown that filtrates from UDN-affected tissues can transmit the disease to healthy fish [5,6]. Early attempts to search for a UDN-associated virus based on transmission and scanning electron microscopy were unsuccessful. The possibly broadest research is needed to answer the above questions and hypotheses and to explain the pathological nature of the disease syndromes. The aim of the study was to clarify the pathogenesis of ulcerative dermal necrosis (UDN)—a disease affecting mostly the skin of salmonid fish in the rivers connected with the Baltic Sea and the Atlantic coasts of France, the British Isles and Ireland since the nineteenth century.

## 2. Materials and Methods

A study of the histopathological as well as the infectious nature of UDN syndromes (searching for bacteria, viruses and/or fungi possibly involved in the development of the disease) has been performed. Virological examinations consisted of two parts: (i) in vivo experiments and (ii) virus screening based on NGS (next-generation sequencing) technology. In vivo experiments were aimed at verifying the possibility of transmission of the pathogen from the UDN-affected fish to healthy fish. The NGS-based studies of nucleic acid samples from the UDN-affected fish tissues were performed using high-throughput sequencing on the Illumina platform followed by bioinformatics analysis. The search for bacteria and fungi involved in UDN was carried out using routine, accredited methods (API tests). In addition, extensive study was carried out using a scanning microscope.

### 2.1. Fish Delivery and Maintenance 

Fish were purchased from the Department of Salmonidae Fish Breeding at the Inland Fisheries Institute (IFI), Rutki, Poland. The purchased fish came from two families (R3 and R4), and they weighed 360–670 g [7]. Before the experiments, the fish were conditioned and acclimated in two pools (with a water capacity of 1100 L/each) for 10 days. During this period, fish were fed twice a day with standard feed intended for the given species (Aller silver 4.5 mm), which covered all nutritional requirements for rearing. The fish were provided with appropriate environmental conditions: oxygenated water > 85% oxygen saturation, flow 2–3 cm/s, temperature 12° and water pH 6.5–8.

The fish were looked after by qualified personnel, including a veterinarian with many years of experience in working with fish. In order to improve the living conditions of sea trout in the water reservoir, pebbles and plastic pipes were introduced, through which the fish were able to flow freely, and the pipes acted as hiding places for them.

### 2.2. In Vivo Experiments

Four experiments were carried out aiming to transmit UDN. 

#### 2.2.1. First Experiment

Each fish received an injection of 0.5 mL of homogenate from the pathologically changed skin of 3 UDN-affected sea trout from the Słupia River collected on 29 October 2020 and stored until then at −80 °C. The homogenate was prepared from 3 pieces of about 1 square cm of skin from 3 fish with UDN symptoms by grinding the tissues in a porcelain mortar with added PBS. The homogenate was then centrifuged at 2000× *g*, filtered through a 0.45 µm filter and divided into 0.5 mL doses. The homogenate was administered intraperitoneally to 20 fish at a dose of 0.5 mL (10 R3 sea trout and 10 R4 sea trout each). Fish from two families (10 pcs.) were placed in separate 600 L aquariums with water flow at a temperature of approx. 12 °C and oxygenation of 9–10 mgO_2_/mL. The control group consisted of R3 and R4 sea trout in the pools. 

#### 2.2.2. Second Experiment

The homogenate was prepared from bulk samples of internal organs (about 1 cubic cm of kidney, spleen and liver tissue each) taken from 3 UDN-positive sea trout from the Słupia River in October 2020, by grinding in a porcelain mortar with PBS added. The homogenate was then centrifuged at 2000× *g*, filtered through a 0.45 µm filter and divided into 0.7 mL doses. The homogenate was used to inject each of 10 R3 sea trout and 10 R4 sea trout. Fish were placed in separate aquariums with a capacity of 600 L with water flow in a closed system at a temperature of approx. 12 °C and oxygenation at the level of 9–10 mgO_2_/mL. One R3 fish did not wake up from sedation and fell asleep. The control group included the same R3 and R4 sea trout injected with PBS only. 

#### 2.2.3. Third Experiment

In August 2021, a homogenate was prepared from 2 pieces of skin (about 1 cm^2^ from two fish with UDN symptoms collected in October 2020 in the Słupia River) by grinding in a porcelain mortar with PBS added. The homogenate was then centrifuged at 2000× *g*, filtered through a 0.45 µm filter and divided into 0.5 mL doses. The homogenate doses were mixed with an equal volume of the steroid. The mixture was administered intraperitoneally at a dose of 1 mL to a total of 10 fish from IFI (5 R3 sea trout and 5 R4 sea trout each). Fish from two families were placed together in a 600 L aquarium with water flow at a temperature of approx. 12 °C and oxygen concentration at the level of 9–10 mgO_2_/mL. The control group consisted of 10 sea trout from the IFI (5 each R3 and R4) placed in a second 600 L aquarium with water flow in a closed system at a temperature of approx. 12 °C and oxygenation at the level of 9–10 mgO_2_/mL after prior intraperitoneal administration of the mixture of equal amounts of PBS and steroid in a volume of 1 mL. 

#### 2.2.4. Fourth Experiment: Cohabitation

In the fourth experiment, the procedure for UDN transmission was based on the model of infection by cohabitation of healthy sea trout from the R3 and R4 families, obtained from the IFI, and individuals caught in the Słupia River in November 2021. Ten sea trout spawners from the Słupia River with clear symptoms of UDN disease were transported in bags with water and oxygen to the aquariums of the National Veterinary Research Institute (NVRI) in Puławy. After prior sedation and injection with methylprednisolone at a dose of 4 mg/fish [8], 5 sea trout with UDN symptoms and 5 healthy fish weighing 380 to 500 g, each from the R3 and R4 families from the IFI, were placed in each of the two pools in a closed system with a capacity of 1000 L. Fish from the R3 and R4 families, which were given an immunosuppressant, were also placed in a separate reservoir as a control group.

### 2.3. Clinical Examination

A clinical examination was performed on the moribund fish. The abnormal clinical symptoms were recorded, while a necropsy was carried out to evaluate the postmortem (PM) lesions in the affected organs.

### 2.4. Sample Collection

Fish were humanely killed using MS-222 and subjected to a necropsy. During the necropsy, samples of all organs showing pathological lesions as well as corresponding control samples from clinically healthy fish were collected and transported to the laboratory (National Reference Laboratory for Fish Diseases at the NVRI in Puławy) as soon as possible in the appropriate conditions (temp. about +4 °C). Tissue samples for histopathological and electron microscopy study were collected into containers with 10% buffered formalin and PBS-buffered 2–4% paraformaldehyde solution with 0.5% glutaraldehyde, respectively. Other fish samples were collected under aseptic conditions, directly frozen in liquid nitrogen and then stored at a temperature of −80 °C for proteomic study.

### 2.5. Histopathological Assessment of UDN Lesions

Sections of pathologically affected and healthy fish tissues (negative control) were collected in containers with 10% buffered formalin and delivered to the laboratory at the NVRI in Puławy. Specimens fixed in formalin were routinely processed in a tissue processor, embedded in paraffin, cut into 5 μm sections and, after staining with hematoxylin and eosin, analyzed by light microscopy (Carl Zeiss Axiolab 5 Germany). Several illustrative photomicrographs were taken from the prepared sections using a digital camera (Carl Zeiss Axiocam 208, Oberkochen, Germany) connected to a microscope.

### 2.6. Scanning Electron Microscopy (SEM)

The investigated material consisted of small skin pieces with and without lesions. The material was fixed in glutaraldehyde (2.5%) and formaldehyde (4%) in 0.1 M sodium phosphate buffer of pH 7.4 for 6–12 h and kept at 4 °C. The tissues were washed twice in sodium phosphate buffer and post-fixed in osmium tetraoxide (1%) for 4 h. The tissues were washed in distilled water and then dehydrated using graded concentrations of ethanol (25%, 50%, 70%, 90%, 99.8%) and acetone. The samples were dried with CO_2_ using the critical point dryer (Quorum Technologies, Polaron CPD7501). Thereafter, they were mounted on aluminum stubs, metalized with gold–palladium ions in a Sputter Coater (Quorum Technologies, Polaron, Newhaven, UK) and observed under a scanning electron microscope (Carl Zeiss QEC GmbH, Oberkochen, Germany). Photos were taken from the most relevant aspects.

### 2.7. Virological Examination

A total of 39 sea trout (*Salmo trutta* m. *trutta* L.) caught in one of the rivers of northern Poland during the migration to spawning were tested for the presence of viruses causing infections in salmonids. To this end, the presence of IHN, IPN, ISA and VHS viruses was studied using accredited methods applied in the fish diseases reference laboratory in Puławy, Poland.

#### 2.7.1. Virus Isolation in Cell Cultures

The following cell lines were used: for IHN and IPN viruses, the EPC (epithelioma papulosum cyprinid) cell line, and for the VHS virus, the BF-2 (bluegill fry) cell line. Cell cultures were prepared in 24-well plates by seeding 2 × 10^5^ cells/mL in fresh medium (Tris-buffered Eagle’s MEM with 10% fetal bovine serum) and culturing at 15 °C. The cells were inoculated with supernatants when 80–100% confluence had been reached. The cultivation was continued until the cytopathic effect t (CPE) was evident. Then, the cell culture supernatants were collected and used for the isolation of viral RNA. Positive samples were frozen at −80 °C and stored until needed.

#### 2.7.2. RNA Extraction

The infected cell culture monolayers were harvested, and total RNA was extracted using an A&A Biotechnology RNA Purification Kit (Gdynia, Poland). Total RNA was purified according to the manufacturer’s instructions, eluted in 100 µL of RNase-free water and stored at −80 °C.

#### 2.7.3. RT-PCR

Reverse transcription of IPNV RNA and amplification of cDNA were carried out by using the commercial one-step RT-PCR kit (EURx, Gdańsk, Poland). A 50 µL aliquot of the reaction mixture was prepared and contained 5 µL of RNA, 2× Master Buffer Mix, Master Enzyme Mix, 10 µM of specific primers and RNase-free water.

Primers were designed by using Primer3Plus software [9], as follows: for VHS [10], for IHN [11] and for IPN [12].

The specificity of the obtained products was verified by sequencing at the Genomed Company, Warsaw, Poland. The products of RT-PCR were detected in 2% agarose gel with Simply Safe.

For the diagnosis of ISA (infectious salmon anemia) virus, the RT-PCR method using the primers described in OIE Manual [13] was applied.

The research material included internal organs of fish (heart, kidney, spleen and liver), from which RNA was isolated. Total RNA was extracted using an A&A Biotechnology RNA Purification Kit (Gdynia, Poland). Total RNA was purified according to the manufacturer’s instructions, eluted in 100 µL of RNase-free water and stored at −80 °C.

The study was conducted using the reverse transcription method. The reaction mixture (45 μL) included 2× reaction mix, starters I and II (10 μM each) 1 μM, superscript II RT/Platinum Taq Mix (5 U/μL)—1 μL and sterile water—17 μL. A total of 5 μL of isolated RNA was added to the mixture prepared in this way. RT-PCR amplification consisted of reverse transcription for 30 min at 50 °C, initial denaturation for 4 min at 95 °C, proper denaturation for 30 s at 94 °C, primer annealing for 30 s at 55 °C, elongation for 1 min at 68 °C and cDNA synthesis (final extension) for 7 min at 68 °C. The products of RT-PCR (155 bp) were detected in 2% agarose gel with Simply Safe.

### 2.8. Bacteriological and Mycological Examination

Sea trout caught during the migration to spawning were tested. For the study, only fish with evident pathological lesions on the skin were selected. All fish were humanely killed using Tricaine Methanesulfate (MS-222) (Aldrich, Wuxi, China) and subjected to necropsy.

The kidney and skin were separated from each fish and homogenized in a sterile phosphate-buffered saline solution (Biomed, Lublin, Poland) in a ratio of 1:1 (*w*/*v*). For bacterial isolation, the samples were inoculated into nutrient agar supplemented with 5% horse blood—BA (Biomaxima, Lublin, Poland), tryptic soy agar—TSA (BioMérieux, Marcy-I’ Etolie, France) and Cytophaga agar—CA. The incubation of BA and TSA was carried out at 27 ± 1 °C for 48–72 h and CA for 5–7 days at 17 ± 2 °C. After growth, the pure bacterial colonies were isolated, Gram stained and biochemically identified using API kits and the VITEK2 system (BioMérieux, Craponne, France). API tests were performed according to the manufacturer’s instructions, and the temperature of incubation was 27 ± 1 °C. Bacteria with a morphology characteristic of the *Flavobacterium* genus were identified based on 16S rRNA gene sequence analysis, as described previously [14].

For isolation of fungi, the homogenates were plated into Sabouraud agar medium (BTL, Łódź, Poland) and incubated at 27 ± 1 °C for 5 days. After growth, pure cultures were isolated from Sabouraud liquid medium and incubated for 2 days at 27 ± 1 °C with shaking. The fungi were observed under a light microscope and identified by the semi-nested PCR method [15]. Total DNA was isolated according to the manufacturer’s instructions using the DNeasy Plant Mini Kit (Qiagen, Hilden, Germany). The conserved ITS (internal transcribed spacer) region of the fungus was amplified, and PCR products were sequenced using a 3730xl DNA Analyzer (Genomed S.A, Craponne, France). Sequences were assembled using the MEGA 10.2.6 software, and phylogenetic analyses were computed with the neighbor-joining method [16].

## 3. Results

### 3.1. In Vivo Experiments

**Figure 1:**
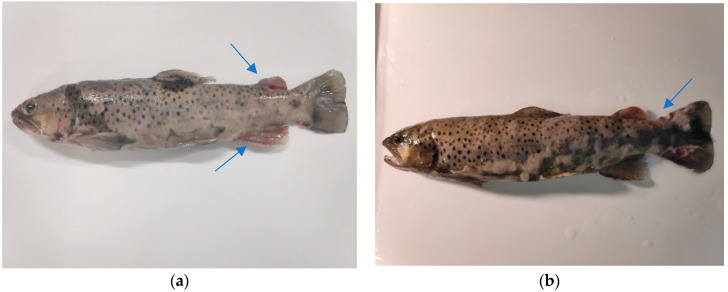
(**a**) Brown trout from Słupia River. (**b**) Experimental fish (brown trout) 14 days after the date of cohabitation experiment. Both fish show lesions in the back and caudal fin. Growing hyphae display a cotton-wool-like appearance. Extravasations (**a**) and erosions (**b**) on the fins are visible.

In total, four experiments were carried out on fish, the aim of which was to induce pathological lesions similar to those observed in natural conditions. Each of these experiments lasted 3 months. The first three experiments relied on the intraperitoneal injection of tissue homogenate obtained from the UDN-affected sea trout into healthy fish. In the first experiment, we used a homogenate of pathologically changed skin, in the second experiment, a homogenate of internal organs (kidney, spleen and liver in equal proportions), while in the third experiment, skin homogenates were also applied, but in addition, the fish were immunosuppressed through injection of DEPO Medrol. All these experiments did not cause skin changes characteristic of UDN in experimental fish, despite the at least 3-month observation period. Only the fourth experiment relying on the cohabitation of the UDN-affected sea trout with healthy fish was successful. As a result, transmission of the disease and the appearance of lesions on the skin of experimental fish characteristic of UDN was observed. The first symptoms of UDN in experimental sea trout subjected to cohabitation with sick fish appeared after 12 days of observation. Within the next week, pathological changes appeared in all cohabited fish (Figure 1). In the following days, samples were picked up from the remaining fish successively. Within the next 2 weeks, the experiment was terminated. There were no symptoms of UDN in the R3 and R4 fish from the control, immunosuppressant-receiving group.

### 3.2. Histopathological Assessment of UDN Lesions

**Figure 2:**
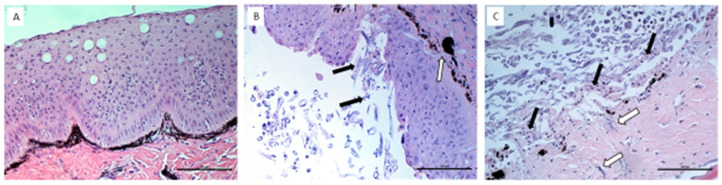
Photomicrographs of the skin from control trout (**A**) and the skin from the trout with ulcers (**B**,**C**). (**A**) Intact dermis and the epidermal layer in the control skin. (**B**) The epidermis is desquamated and disrupted by the fungal hyphae (black arrow), which reaches the subepidermal part of the skin (white arrow). (**C**) The epidermal layer is completely desquamated, the dermis is disrupted by the fungal hyphae (white arrows) and the superficial dermal layer is necrotic and covered with cellular debris (arrows). Scale bar = 100 µm.

The H&E-stained skin samples from 10 different UDN-positive fish were evaluated under a light microscope (Carl Zeiss Axiolab 5). Different pathological lesions were observed. The most frequent were focal exfoliation of the epidermis, focally longitudinal mycelia penetrating between the epidermal cells and into the dermis, multifocal complete epidermal loss and necrosis within the dermis, moderate infiltration of lymphoid cells in the dermis, multifocal epidermal defects and necrosis within the dermis. The above lesions were not observed in each fish. Sometimes, only a few of them were present in individual fish (Figure 2).

### 3.3. Bacteriological and Mycological Examination

**Figure 3:**
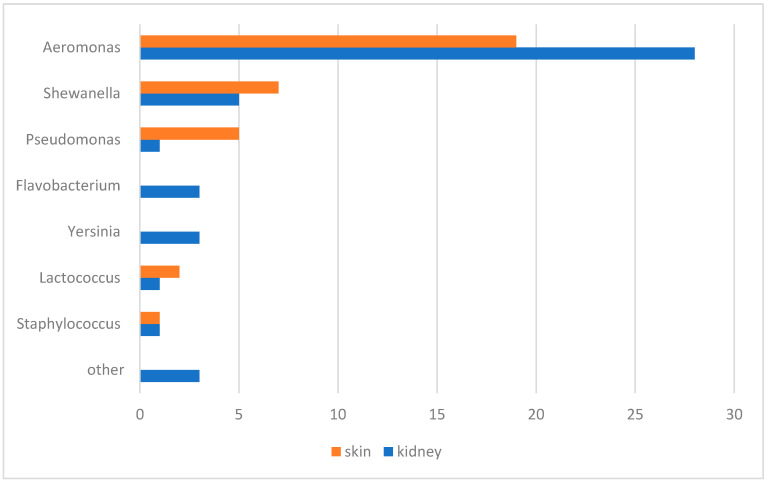
Number of bacteria isolates in samples of skin and kidney.

**Figure 4:**
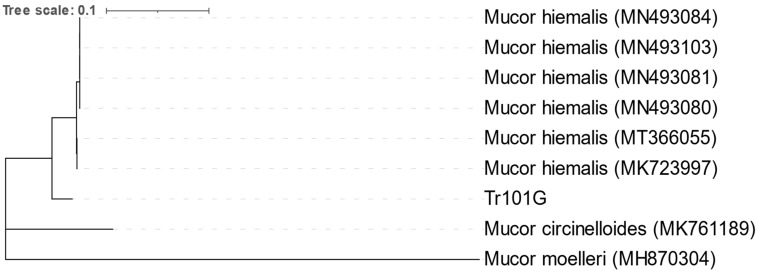
Analysis of partial ITS gene of fungi isolated from skin.

Bacteria were isolated only from fish with health disorders. The following bacteria were isolated from kidney samples: *Aeromonas* spp., *Shewanella* spp., *Pseudomonas* spp., *Flavobacterium* spp., *Yersinia ruckeri*, *Acinetobacter lwoffii*, *Ewingella americana*, *Serratia* spp., *Lactococcus* spp. and *Staphylococcus warnerii* (Figure 3). From skin lesions, *Aeromonas* spp., *Shewanella* spp., *Pseudomonas* spp., *Lactococcus piscium* and *Staphylococcus warnerii* were detected. In addition, fungi morphologically similar to the Mucor genus were isolated from skin lesions. The greyish-white colonies were 3–4 cm in size and looked similar to cotton candy. Based on phylogenetic analysis of the partial ITS region, the isolated fungi were most similar to the *Mucor hiemalis* species (Figure 4).

The neighbor-joining phylogenetic tree was constructed based on the partial nucleotide sequence of the ITS gene, which was compared with the sequence available in GenBank (MN493080 isolate WDH from *Perca fluviatilis*, MN493081 WJQ from *Hypophthalmichthys molitrix*, MN493084 JY06 from *Carassius auratus*, MN493103 DH from *Oncorhynchus mykiss*, MK723997 C5M5F9_Wh6 from *Oncorhynchus mykiss* and MK761189 C8M8F10 from *Oncorhynchus mykiss*) [17]. The evolutionary distances were computed using the p-distance method [18]. There were a total of 284 positions in the final dataset.

### 3.4. Scanning Electron Microscopy (SEM)

**Figure 5:**
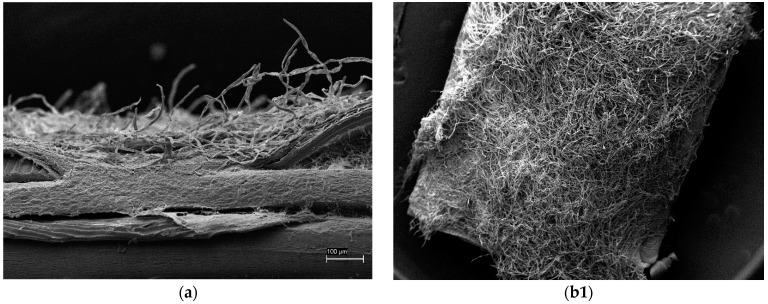

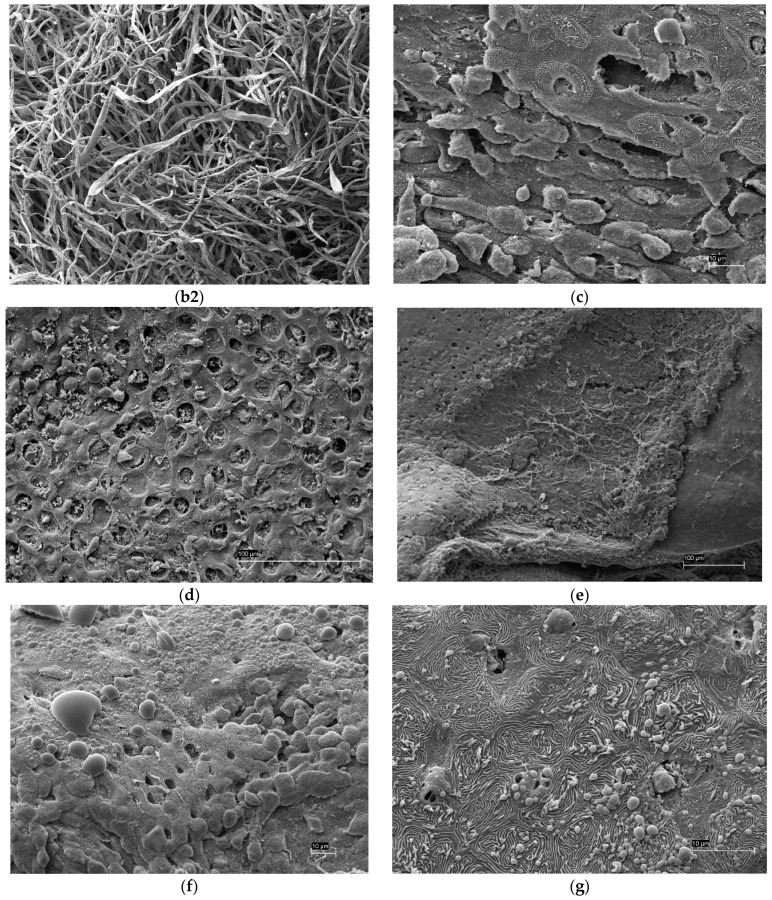
Scanning electron microscopy (SEM) of the skin sections of UDN-affected fish. (**a**,**b1**,**b2**) Very numerous mycelia were visible. The surface of the skin was completely covered with mycelium; therefore, the description of changes in epidermal cells was not feasible. (**c**) Locally, a lack of epidermal integrity and detachment of epidermal cells was observed. Detachable epidermal cells sometimes show damage/necrosis, and sometimes, cells with intact microridges are visible. (**d**) Visible in high-density traces of mucous cells—characteristic round holes. The cells around them have a changed surface—no microridges. (**e**) In some places, a significant reduction in the thickness of the epidermal layer and a developing mycelium was observed. (**f**,**g**) Visible blistering of epidermal cell microridges and mucous cell secretion accompanies epidermal damage/defects.

Skin sections collected from pathologically changed areas revealed that the skin was very thin, and the epidermis layer was barely noticeable or absent. Very numerous mycelia were visible. The surface of the skin was completely covered with mycelium; therefore, the description of lesions in epidermal cells was not feasible. Locally, a lack of epidermal integrity and detachment of epidermal cells was observed. Detachable epidermal cells sometimes showed damage/necrosis, and sometimes, cells with intact microridges were visible. In some places, a significant reduction in the thickness of the epidermal layer and a developing mycelium was observed. Damage or defects of the epidermal layer were accompanied by vesicle formation in the epidermis cells and visible discharge from mucous cells (Figure 5).

## 4. Discussion

The experiments 1–3 revealed that a single injection of the pathological material collected from the UDN-affected fish into healthy fish is not effective for transmission of the disease. In contrast, successful transmission was reached when the change from single injection to cohabitation of healthy fish with the UDN-affected fish was applied. This means that prolonged exposure of the fish sensitive to infection is much more efficient in causing disease than the single injection of the causative pathogen. Such a scenario seems rational also because various pathogens, not only bacteria but also fungi, are among the factors responsible for pathological changes. This confirms the complex etiology of UDN. The key factor that seems to play a role in the pathogenesis is a significant weakening of the general immunity of diseased fish, especially in relation to infectious agents present in the external environment, which is associated with changes in important immunological parameters. The basis of this tendency is, among others, a reduction in the number of lymphocytes in wild populations of sexually mature brown trout during the spawning period [19]. The population of immunoglobulin-producing cells in the blood, head, kidney and spleen is also reduced [20,21]. A decrease in the number of immunoglobulin-producing cells was observed during the rainbow trout spawning season regardless of the length of the day and water temperature, which suggests that the main factor causing these changes was the higher level of sex steroids [22]. It is worth noting that the UDN problem concerns fish that are spawning or have already spawned. So far, we have not observed a situation, in which the UDN problem affects sea trout in other periods of its life. The mentioned weakening of the immunity of fish during the spawning period is related to the need to travel long distances from the place of normal existence to the spawning ground, the occurrence of obstacles along the migration route and the shortage of food [23]. Moreover, our observations show that fish during spawning migration show a significantly reduced interest in feeding. Another phenomenon observed during this journey of anadromous fish is the increasing burden of pathogens in migrating fish. This phenomenon has been observed, for example, in migrating adult Chinook salmon in the upper Willamette River Basin [24]. Changing the environment from saltwater to freshwater creates new threats related to the exposure of migrating fish to new pathogens characteristic of freshwater. The fish’s immune system must cope with new pathogens in a new environment. If unsuccessful, this may have a lethal effect on the fish. R.A. Tripp et al. [25], trying to explain the mechanism of the reduced activity and inhibition of immunoglobulin production by B lymphocytes, measured the transcript levels of selected immunosuppressive cytokines and found no significant changes during migration. On this basis, they concluded that the weakened immune function in spawning salmon is not caused by pathogen infection or cytokine-mediated immune suppression but rather is related to the reduced energy reserves or hormonal changes accompanying spawning. It is known, for example, that during the spawning period, the level of cortisol increases significantly, which, by inhibiting the production of lymphokines, inhibits the production of antibodies by lymphocytes. The role of the general weakening of fish immunity resulting from long migration in the pathogenesis of UDN is confirmed by the negative results of the first three experiments, in which the disease was not transferred after a single injection of pathogens isolated from the lesions. Normally, disease transmission should occur if the pathogen present in the injection is the obligatory causative agent. However, this did not happen, which shows that other conditions are needed to obtain the expected outcome. Firstly, it seems that, for the effective transmission of the disease, a longer exposure (preferably several days) as well as concomitant immunosuppression is required. The application of artificially created appropriate conditions by using a known immunosuppressant (DEPO Medrol) and prolonged exposure of the experimental fish to a potential pathogenic agent resulted in disease transmission.

The key role of immunosuppression in the pathogenesis of UDN manifests itself especially during the spawning migration. Spawning is an extremely stressful period in the life of fish, which is accompanied by profound changes in the body’s physiology. This phenomenon also extends to the skin. There is extensive literature confirming that fish skin is the first line of defense against various pathogens [26,27,28,29]. Skin mucus is an important part of the innate immune mechanism in fish and provides a physical and chemical barrier against pathogens, thus playing an important role in fish health. UDN becomes a problem when fish migrate to spawn. Fish are very weakened by long migration and reduced food intake during this period related to focusing attention on reaching the spawning ground and laying eggs. Such behavior has consequences such as reduced immunity and increased susceptibility to environmental pathogens. Immunodeficiency related to nutritional shortage is well documented in the scientific literature [30,31,32,33]. It also has a negative impact on maintaining skin homeostasis and defense functions. Changes in skin physiology may be of particular importance in the context of the etiology of UDN. There are many publications available on the function of the epithelial barrier in preventing bacterial infections in salmonids and other fish species [34,35].

The histopathological examination confirmed a variety of changes in the skin of the UDN-affected fish compared to healthy fish. First of all, the vast majority of the affected fish showed significant thinning of all layers of the skin. The severity of the changes depended on the particular fish. The changes differed, but they were usually focal. In the case of the most affected fish, the histological structure of the skin was completely destroyed, and numerous fungi and bacteria were shown to be present. The bacterial flora consisted mainly of commensal and saprophytic strains, but the presence of pathogenic bacteria, e.g., bacteria from the *Shewanella putrefaciens* group or fungi, e.g., *Mucor hiemalis*, was also confirmed. The appearance of pathogenic fungi seems to be the last stage of the disease and is usually preceded by complete disintegration of the skin structure in the places where they appear. More insight into the structure of the affected skin was enabled by the use of a scanning microscope. The study revealed a series of significant changes within this tissue that contribute to the development of the disease. In the literature, such detailed studies of bacteria-infected fish are scarce. To identify the mechanism of disease progression, it is crucial that the problem concerns anadromous fish. The change in the environment from salty to freshwater during the migration to spawning has an impact on both the histological structure of the fish skin and the physicochemical properties of the mucus. Numerous studies have reported that spawning migration connected with changes in salt content, pH, temperature and ion concentration affects the size and reduces the density of mucus cells in fish [17,36,37,38]. The skin of anadromous fish shows incredible plasticity in contact with changing environmental conditions. In addition to the parameters already mentioned, the reproductive migration of fish causes modifications of the chemical composition of mucus. An example is anadromous fish from Lake Van, Turkey [38], which increases the secretion of neutral glycoconjugates in the mucus when migrating to spawn in freshwater and return to secrete sulfated and acidic glycoconjugates in the waters of Lake Van. The concentration of the basic mineral components of the mucus, such as sodium, potassium chloride, magnesium and chlorine ions, also changes dramatically. It is also worth mentioning the changes in the composition of the microflora encountered by fish migrating from the marine environment to freshwater, which, taking into account the physiological modifications already discussed, especially significant thinning of the skin, seems to be an important factor in the pathogenesis of UDN [39]. The changes in the composition of mucus, as well as in the number of mucous cells in the skin, associated with the transition from the marine environment to freshwater, are accompanied by modifications in the structure of the epidermis in the form of damage or loss of microprotrusions. The preserved microprotrusions are sometimes characterized by a loss of continuity or fragmentation. The rearrangement of the layout of microprotrusions can be caused by several external and internal factors. Such rearrangements have been described in the course of bacterial infections of fish, including *Flavobacterium psychrophilum* infection causing cold water bacterial disease in the ayu *Plecoglossus altivelis* [40] or *Aeromonas hydrophila* infection in the common carp *Labeo rohita* [41], changes in salinity [42] or stress caused by disease organisms such as fungi and bacteria [43]. Fish epidermal mucus is a rich source of antimicrobial peptides as a result of the permanent contact of the fish with the surrounding environment. Among them, numerous antimicrobial peptides, i.e., lysozyme, alkaline phosphatase, cathepsins, lectins and proteases, as well as amphipathic peptides acting as detergent-dissolving membranes of microorganisms, deserve special attention. A detailed review can be found in [44]. Late summer or autumn is a period when the bacterial load in water increases significantly due to favorable temperature conditions. Usually, the first reaction to a skin infection is increased secretion of mucus by goblet cells, which, thanks to its bactericidal properties, protects the skin against pathogenic microorganisms. However, constant confrontation with pathogens present in the surrounding environment depletes mucus production and facilitates successful invasion. On the one hand, it is also an explanation for unsuccessful attempts to induce UDN through a single injection of pathogenic material and a response to the possible mechanism of disease development, which is the need for long-term exposure of fish (weakened by long spawning migration) to numerous pathogens in the surrounding environment (several days of cohabitation).

## 5. Conclusions

In conclusion, our study showed a complex etiology of the disease. Its final stage is characterized by severe complications of an infectious nature. Not one but many pathogens, both bacterial and fungal, have been identified. The disease is therefore polyetiological in nature. Additionally, predisposing factors include the reproductive period with its accompanying hormonal changes, the change in the environment from salt- to freshwater, the effort associated with the long migration to spawning and probably the lack of food caused by focusing on the main goal of this migration, which is spawning.

## Data Availability

Data are contained within the article.
